# RETRACTION: The Effect of Two Surgical Modalities for the Treatment of Subtrochanteric Fractures of the Femur on Postoperative Wound Complications in Patients: A Meta‐Analysis

**DOI:** 10.1111/iwj.70388

**Published:** 2025-03-21

## Abstract

**RETRACTION:** ChenB. , LiS. , ZengF. , duanH. , and LuM. , “The Effect of Two Surgical Modalities for the Treatment of Subtrochanteric Fractures of the Femur on Postoperative Wound Complications in Patients: A Meta‐Analysis,” International Wound Journal 21, no. 3 (2024): e14421, 10.1111/iwj.14421.37931601 PMC10895200

The above article, published online on 06 November 2023, in Wiley Online Library (http://onlinelibrary.wiley.com/), has been retracted by agreement between the journal Editor in Chief, Professor Keith Harding; and John Wiley & Sons Ltd. Following an investigation by the publisher, all parties have concluded that this article was accepted solely on the basis of a compromised peer review process. The editors have therefore decided to retract the article. The authors did not respond to our notice regarding the retraction.

**RETRACTION:** B. Chen , S. Li , F. Zeng , H. duan , and M. Lu , “The Effect of Two Surgical Modalities for the Treatment of Subtrochanteric Fractures of the Femur on Postoperative Wound Complications in Patients: A Meta‐Analysis,” International Wound Journal 21, no. 3 (2024): e14421, 10.1111/iwj.14421.37931601 PMC10895200

The above article, published online on 06 November 2023, in Wiley Online Library (http://onlinelibrary.wiley.com/), has been retracted by agreement between the journal Editor in Chief, Professor Keith Harding; and John Wiley & Sons Ltd. Following an investigation by the publisher, all parties have concluded that this article was accepted solely on the basis of a compromised peer review process. The editors have therefore decided to retract the article. The authors did not respond to our notice regarding the retraction.

